# Correction: Development of wound healing scaffolds with precisely-triggered sequential release of therapeutic nanoparticles

**DOI:** 10.1039/d0bm90105a

**Published:** 2020-11-23

**Authors:** Tauseef Ahmad, Sean McGrath, Catherine Sirafim, Ronaldo J. F. C. do Amaral, Shin-Loong Soong, Renuka Sitram, Shifaa Turkistani, Francesco Santarella, Cathal J. Kearney

**Affiliations:** Tissue Engineering Research Group, Dept. of Anatomy and Regenerative Medicine, Royal College of Surgeons in Ireland (RCSI) Dublin Ireland; Advanced Materials and Bioengineering Research Centre (AMBER), RCSI and Trinity College Dublin Dublin Ireland; Trinity Centre for Bioengineering, Trinity College Dublin Dublin Ireland; Department of Biomedical Engineering, University of Massachusetts Amherst USA ckearney@umass.edu +1 413-545-1073

## Abstract

Correction for ‘Development of wound healing scaffolds with precisely-triggered sequential release of therapeutic nanoparticles’ by Tauseef Ahmad *et al.*, *Biomater. Sci.*, 2020, DOI: 10.1039/d0bm01277g.

In the original manuscript, an author's name was spelled incorrectly. The correct spelling is “Shifaa Turkistani”, as shown above.

The Royal Society of Chemistry apologises for these errors and any consequent inconvenience to authors and readers.

## Supplementary Material

